# Proposed Criteria for Constipation in Palliative Care Patients. A Multicenter Cohort Study

**DOI:** 10.3390/jcm10010040

**Published:** 2020-12-25

**Authors:** Tomasz Dzierżanowski, Philip Larkin

**Affiliations:** 1 Laboratory of Palliative Medicine, Department of Social Medicine and Public Health, Medical University of Warsaw, 02-007 Warsaw, Poland; 2 Palliative and Supportive Care Service, Lausanne University Hospital and University of Lausanne, CH-1010 Lausanne, Switzerland; Philip.Larkin@chuv.ch

**Keywords:** constipation, palliative care, diagnosis, laxatives

## Abstract

Although constipation is one of the most frequently reported symptoms in palliative care, there is no widely accepted definition of constipation and none suitable for patients unable to self-report or express symptoms. This study aimed to verify the objective and subjective symptoms of constipation to develop a diagnostic algorithm for constipation, which is also feasible in unconscious patients. In a pooled analysis of two observational studies, 369 out of 547 adult end-stage cancer patients met the inclusion criteria. The patient-reported difficulty of defecation correlated with observable measures, such as days since last bowel movement, and frequency of bowel movements. Difficulty became at least moderate when there were no bowel movements for ≥2 days, or the frequency of bowel movements was ≤3 per week. The diagnostic algorithm, comprising these three symptoms offers a simple, rapid, and comprehensive tool for palliative care, independent of the patient’s state of consciousness. A clinical trial is necessary to confirm its validity and usefulness.

## 1. Introduction

The prevalence rate of constipation varies from 40% to 90% in palliative care patients [1], highly dependent on the definition of constipation, its objective and subjective metrics, and the discriminating cut-off values used [2,3,4]. The available definitions of constipation include (1) the patient’s subjective perception, reflecting the level of their discomfort and changes in bowel movements (BM) pattern, and (2) observable (measurable) symptoms, such as the frequency of defecation [4]. Bowel movement and defecation are commonly used interchangeably to describe the act of excreting feces.

There is no widely accepted definition of constipation in palliative care patients despite its high prevalence. Definitions used in research are diverse and contradictory with each other [4]. The breadth of definitions in current literature reveals that there is no single set of diagnostic criteria, and different combinations of patient-reported and objective measures have been identified [4]. There are also research studies in which constipation was diagnosed through clinical judgment, without specific diagnostic criteria, or without a clear definition [4]. Furthermore, only one out of 20 definitions of constipation identified in this review included a patient’s self-assessment as the main reason to diagnose constipation, which contrasts with the subjective nature of the symptom [4]. In a multicenter cross-sectional survey, constipation varied 6-fold, from 7%, if diagnosed based on the frequency of BM only, to 43%, when patient-reported perception was also considered [2]. Patient-reported assessment should be considered superior, or at least equal to, frequency of BM, as it reflects a patient’s health-related quality of life, the main goal of palliative care [4]. Beyond this, 50% of patients understand constipation differently from their physicians, and the physician should ensure that this misunderstanding is avoided [5,6].

In one study, 42.4% of patients at the time of referral to palliative care had altered bowel scores expressed through a numeric rating scale (NRS) (0–10) [7]. In this scale [0—no symptom and 10—extremely severe symptom], the values 1–3 usually refer to mild intensity [8]. Though small, any deviation from the patient’s typical pattern of BM may lead to constipation. However, not all deviations from zero automatically mean constipation, particularly if the scale is based on unidimensional judgment. It is, therefore, necessary to find a discriminating cut-off value to avoid over-diagnosis. For instance, one of the commonly used tools, the Bowel Function Index (0–100) (BFI), discriminates constipated from non-constipated patients at ≥ 28.8 value with 95% confidence [9].

Constipation is challenging to treat in palliative care patients. Once the normal bowel pattern is altered, there can be a concomitant deterioration in health-related quality of life, impaired effectiveness of symptomatic therapy, often associated with incremental costs. Therefore, if the intensity of the patient-reported perception of constipation is moderate to high, a diagnosis of constipation is most likely, and a clinician should undertake appropriate measures [10,11].

A significant proportion of palliative care patients have dementia, delirium, agitation, or confusion [12] and cannot express their subjective assessment. In unconscious patients, constipation can remain a clinical problem, as it may easily be overlooked by clinicians, even when the patient needs a clinical intervention. In such cases, only observable criteria can be assessed by the caregiver and offer the only guide on whether to intensify or adjust laxative management. However, in real terms, a proxy report may not express the real extent of patient experience and suffering due to this symptom.

Opioid-induced constipation (OIC) is a frequent problem in palliative care [1]. The Rome IV criteria definition of OIC has not been validated in palliative care patients and might be used only in patients with a longer life expectancy, as it requires the observation of several acts of defecation [13]. Most of the definitions of constipation used in palliative care refer to a reasonably shorter observation period (7 or 14 days) due to short life prognostication.

Taking all the above into consideration, there is a need for more tangible metrics that would help the decision-making process on laxative intervention or augmentation of preventive measures in palliative care. The criteria should be easy to use, rapid, based on recent history (i.e., previous seven days), and include a patient’s assessment if possible. They should also be independent of the patient’s consciousness or ability to express or report a symptom to enable a timely clinical response. Eventually, they should take account of both the diagnosis of constipation and the severity of its symptoms.

Based on previous research [14,15], we identified four symptoms that seemed possible candidates for such criteria: the difficulty of constipation, necessity of laxatives, days since the last BM, and frequency of BM. Consequently, this study aimed to address the following clinical questions:

(1) In adult palliative care cancer patients, what is the correlation between patient-reported assessment of constipation (difficulty of defecation) and the observable criteria of constipation: frequency of BM, days since the last BM, and the necessity of laxatives?
(2) What are the cut-off values of the observable diagnostic criteria for constipation defined as moderate to severe difficulty of defecation in adult palliative care cancer patients?

## 2. Materials and Methods

### 2.1. Data

The data from two multicenter observational studies were merged for a pooled analysis to attain a large balanced sample of inpatient and outpatient patients, representative of the adult palliative care population. Both studies were performed in adult patients consecutively admitted to palliative care units in Poland in 2010-11 (Study 1) [14] and 2018-19 (Study 2) [15], following approval by the Ethical Committee of the Maria Sklodowska-Curie Institute—Oncology Center in Warsaw (84/2009) and the Ethical Committee of the Medical University in Lodz (RNN/280/18/KE). The same structured questionnaire and the same data collection methodology were used in both studies, which allowed for merging the results.

The questionnaires in both studies included the following items:

1. Demographic data.
2. Eastern Cooperative Oncology Group (ECOG) performance status.
3. Bowel symptoms in the last seven days; reported by a patient with the aid of a caregiver:
  - days since the last defecation [days],
  - the frequency of BM [days per week with BM],
  - the difficulty of defecation (or disturbed ease of defecation) on (0–4) scale, where 0—no difficulty (“normal defecation”), 1—mild (“rather normal”), 2—moderate, 3—significant/often, 4—extreme difficulty/always (assessed by a patient only),
  - stools too small on (0–4) scale, where 0—normal stools, 1—from time to time (mild intensity), 2—quite often (moderate intensity), 3—very often, 4—always,
  - stools too hard on (0–4) scale (as above),
  - the feeling of incomplete bowel movement on (0–4) scale, where 0—no symptom, 1—mild intensity/sometimes, 2—moderate intensity/quite often, 3—significant intensity/very often, 4—extreme intensity/always (assessed by a patient only),
  - straining to try to pass bowel movements, on (0–4) scale (see above),
  - the necessity of laxatives use on (0–4) scale, where 0—no laxatives used, 1—from time to time (occasionally), 2—often used, 3—bowel movements only after the use of regular laxatives, 4—bowel movements only after an enema or manual stool evacuation.
4. The modified BFI [9] and the Patient Assessment of Constipation Symptoms (PAC-SYM) [16] questionnaires—only in Study 1.
  The modified BFI consists of three questions assessed on a (0–4) scale:
  1. ease of defecation (0 = easy/no difficulty; 10 = severe difficulty),
  2. feeling of incomplete bowel evacuation (0 = not at all; 100 = very strong),
  3. personal judgment of patient regarding constipation (0 = not at all; 10 = very strong) during the last seven days according to the patient’s assessment.

The modified PAC-SYM tool consists of 12 symptoms assessed on a (0–4) scale, where 0 means absent and 4—very severe.

5. The medications used in the last seven days.

Items c–h described in point 3 above reflect the patient’s subjective judgments and their perception of a symptom’s intensity. For any symptom/metrics, an intensity value of ≥2 (moderate or higher) on (0–4) scale was regarded as pathognomonic (diagnostic).

### 2.2. Eligibility Criteria

Records of patients admitted to palliative care service aged ≥ 18 years with a cancer diagnosis were used. The exclusion criteria were:

(a) colostomy,
(b) diarrhea,
(c) bowel obstruction,
(d) the use of prokinetics (i.e., metoclopramide, itopride),
(e) the use of acting mu-opioid receptor antagonists (methylnaltrexone, oxycodone/naloxone controlled-release tablets) during the 7-day observation period.

### 2.3. Statistical Analysis

Frequency analysis was performed using the Chi-square exact test. For non-parametric data, the Mann-Whitney U test was applied. The correlation between non-parametric (ordinal numeric) measures was tested with Spearman’s rank coefficient. *p* values less than 0.05 were considered statistically significant. The exact *p*-value has been presented whenever possible, and a 95% confidence interval (95% CI) is applied.

The analysis was performed in Statistica 13.3 (TIBCO Software Inc., 2017, http://statistica.io, Palo Alto, CA, USA).

## 3. Results

Structured data sets were collected from 547 patients (286 from Study 1 and 261 from Study 2) in 12 centers across Poland. A balanced representation of end-stage cancer patients regarding sex, age, primary tumor diagnosis, performance status, and type of palliative care service (inpatient, home care, or outpatient clinic) was identified. After a quality check process, 178 (33%) cases were withdrawn due to repeated assessment for the same patient or any risk of possible bias, i.e., incomplete or illegible personal data, assessments discordant with a patient’s record, including all 23 reported by a person for whom we found three incompatible questionnaires. Overall, 369 patients were eligible for further pooled analysis. In both study groups, patients were treated with laxatives in a similar way (Table 1).

### 3.1. Demographics

Demographic data are presented in Table 1. The mean age was 68 years (range 26–94), with equal representation of men and women. Forty-two percent were hospice, 31%—home care, and 26%—ambulatory patients. Most (80%) had non-gastrointestinal malignancies (most common: lung 25.7%, breast 11.4%, prostate 7.6%), 6.5%—colorectal, and 12%—other gastrointestinal cancer diagnosed.

The study groups did not differ from each other regarding age, and sex. In Study 1, most of the patients were inpatient, while in Study 2, most were home care patients, which enabled a more balanced representation of these subpopulations. Patients in inpatient hospices had a statistically worse performance status than those receiving at-home care or in palliative ambulatory patients (*p* < 0.05). There was a positive correlation between ECOG performance status and age. The older the patient, the worse their status.

Of all patients, 61% received strong opioids. The mean oral morphine equivalent of opioids (OME) was 99 mg/day, but there were statistically significant differences between Study 1 (56% of patients, the mean OME 87, the median OME 60 mg/day) and Study 2 (71% of patients, the mean OME 113, the median OME 95 mg/day); *p* = 0.004. This reflects the tendency towards increasing opioid analgesics use in Poland from 2011–2019 [17].

Diet modification to ease BM was implemented in 45% of patients. The same proportion of patients had used oral laxatives. In terms of rectal interventions, 29% used suppositories (bisacodyl, glycerol, or both), 12% of patients received an enema, and in 5% of patients, manual stool evacuation was performed in the last seven days.

### 3.2. The Symptoms of Constipation

Table 2 presents the mean and median values of the assessment of symptoms of constipation, including the 12 items composing the PAC-SYM tool, on a (0–4) scale, and BFI on a (0–10) scale. On average, the last BMs were reported 2.4 days before the assessment (median 2). However, 29.3% of patients had not defecated for at least four days. The mean frequency of BM was 2.7 per week (median 2). The average patient reported at least moderate difficulty of defecation (mean 2.2, median 2), and the necessity of laxatives as occasional to frequent (mean 1.5, median 1).

**Figure 1:**
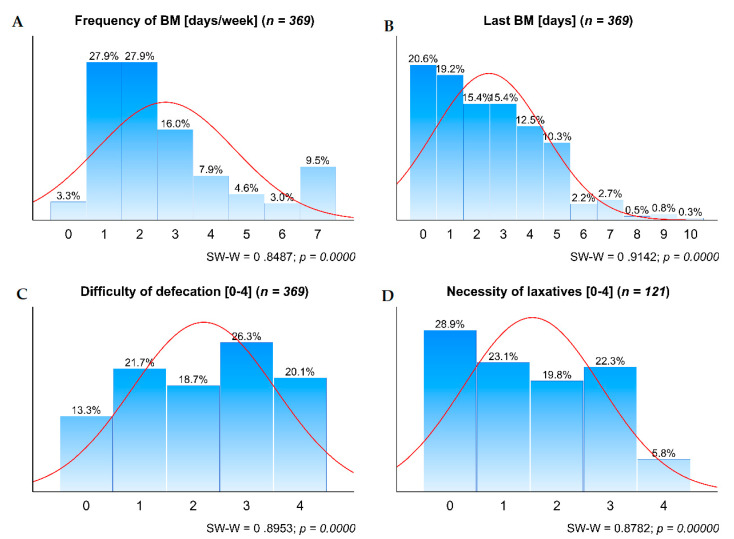
Distribution of the four symptoms of constipation. **A**. Frequency of BM; **B**. Last BM; **C**. Difficulty of defecation; and **D**. Necessity of laxatives. *BM—bowel movements; SW-W—Shapiro-Wilk test for normality*.

These four symptoms were not normally distributed (Figure 1). The graphs (Figure 1) reveal the scale of impairment of BM in palliative care patients. Few patients had BM every day or almost every day, which is regarded as a normal BM pattern. Only 40% had a BM on the assessment day or the day before, and only 13%—without any difficulty. It is noteworthy that less than 30% of patients did not see any need for laxatives.

The mean BFI score was 4.5, which points out that on average, the patients were moderately constipated. The mean intensity of constipation in the PAC-SYM score was lower, with a value of 1.3, where 1 in (0–4) scale means mild intensity. Incomplete BM, stools that were too hard or too small, and straining were frequently reported by patients. On the other hand, certain items, such as cramps, rectal burning, or rectal bleeding, were relatively rare. Although the mean values and their interpretation differed between these two diagnostic tools, they appeared well correlated with each other (*r* = 0.74; *p* < 0.0001).

### 3.3. The Correlation between the Patient-Reported and Observable Symptoms of Constipation

As listed in Table 3, the frequency of BM was negatively correlated with ECOG performance status. The worse the performance status, the less frequent BM were observed (*p* < 0.001).

The two objective measures (time since the last BM and frequency of BM) and the patient-reported difficulty of defecation were correlated with each other (*p* < 0.001) with a logical direction. The necessity of laxatives use, the second patient-reported symptom, appeared associated with the difficulty of defecation (*p* < 0.001) and the frequency of BM (*p* < 0.05) but not with the time since last BM.

BFI strongly correlated with the time since last BM, the frequency of BM (negatively), and particularly the difficulty of defecation for which the Spearman’s correlation coefficient was 0.94 (*p* < 0.001). The relation of BFI and difficulty of defecation was linear with high goodness-of-fit (R-squared = 0.87; *p* < 00001). The mild difficulty of defecation (the mean score 1 on (0–4) scale) corresponds to the mean BFI 2 on (0–10) scale, and moderate (the mean score 2) corresponds to the mean BFI 4.5.

PAC-SYM overall score, as well as the abdominal, rectal, and stool sub-scores, and all the symptoms in the tool correlated with the last BM, frequency of BM, and difficulty of defecation. They also correlated with the necessity of laxatives for the items for which statistical analysis was feasible.

### 3.4. The Cut-Offs for Objective Criteria for Moderate to Severe Difficulty of Defecation

On average, patients assessed the difficulty of defecation as moderately disturbed when the last BM took place two days previously and the frequency was no more than three times per week. However, when the frequency of BM decreased below three per week, the patient-reported difficulty of defecation became severe mean > 2.7 (95% CI 2.6–2.8). The necessity of laxatives was also linearly correlated with the difficulty of BM. Even for the occasional use of laxatives (1 in (0–4) scale), the difficulty of BM, on average, exceeded a moderate level (2.4; 95% CI 2.1–2.6).

Therefore, with the assumption that the intensity of a symptom of constipation assessed as moderate or higher is diagnostic, the following possible criteria for diagnosing constipation were proposed:

1. Difficulty in defecation of ≥ 2 on (0–4) scale (moderate to extreme).
2. Any use of laxatives reported by a patient as necessary to induce BM.
3. Last BM ≥ 2 days.
4. Frequency of BM ≤ 3 per week.

In a post hoc analysis, 84% of patients met at least one of these criteria (Table 4). After withdrawing the necessity of laxatives from the set, the result did not change significantly.

### 3.5. Opioid-Induced Constipation

There were no statistically significant differences for any results between subgroups of patients treated with opioid analgesics and those not taking opioids.

## 4. Discussion

The main finding of this study is that the patient-reported criteria for constipation (the difficulty of BM, the necessity of laxatives) and those which are observable (the frequency of BM, days since last BM) are highly correlated with each other and equally indicative of constipation. In our opinion, subjective patient’s judgment (i.e., the difficulty of defecation, BFI) is more important than objective metrics (frequency of BM, days since the last defecation), as it impacts a patient’s quality of life. However, in patients with dementia or impaired ability to communicate, patient-reported symptoms cannot be obtained. In such cases, the only way is to base decisions on observable criteria, such as frequency of BM or days from the last defecation.

Noteworthy, the necessity of laxatives did not correlate with days since the last BM. A possible explanation is that some patients received laxatives preventively, e.g., together with the opioid therapy. Therefore, laxatives were not used as a rescue medication, and the need for them did not increase along with days since the last BM. The second option, “occasional” and “frequent” use were neither precise nor distinctive.

As mentioned above, BFI is a valuable diagnostic tool, and with the cut-off value of 28.8 [9], constipation is diagnosed in 66.3% of patients [3]. However, this tool may be applied only in conscious and able to self-report patients. The patient-reported assessment of the difficulty of constipation and BFI appeared highly correlated in our study. In the light of that, a simple question on the difficulty of defecation alone seems sufficient for assessing subjective aspects of constipation and equally useful to BFI with high sensitivity. Another finding from a pilot phase of Study 1 was that the numeric analog scale (0–100) for BFI did not offer any greater value over a simple numeric rating scale (0–10), which was equally reported in other review papers [18]. In conclusion, simple scales should be used in daily practice, as they are easy to use, can be repeated, and valid.

All the PAC-SYM items, which are patient-reported symptoms, correlated with BM’s frequency, last BM, and difficulty of defecation, although such symptoms as incomplete BM, stools too hard or too small, and straining seem to be more important. In the previous attempt for cross-cultural adaptation, PAC-SYM appeared too complex or too time-consuming for practitioners involved in the study, and none of the assessing physicians continued using these regularly after the study [14].

**Figure 2:**
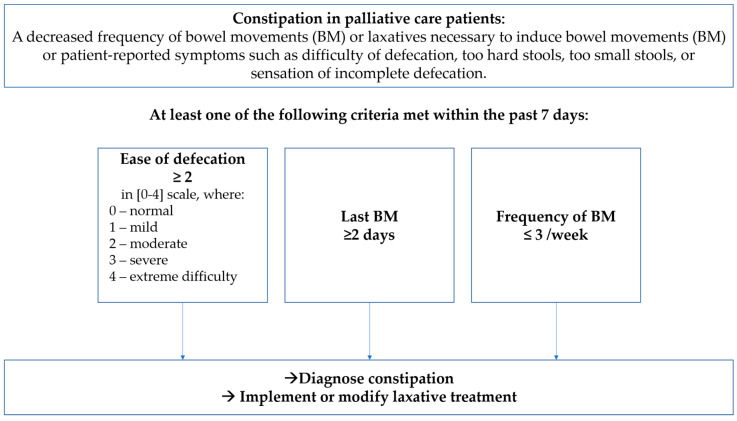
The proposed algorithm ELF-7 for the diagnosing of constipation.

Based on these findings and needing further validation, we propose a simple algorithm for diagnosing constipation in palliative care patients regardless of their ability to self-report, as presented in Figure 2. We propose the following definition of constipation in palliative care patients: “A decreased frequency of bowel movements (BM) or laxatives necessary to induce bowel movements (BM) or patient-reported symptoms such as difficulty of defecation, too hard stools, too small stools, or sensation of incomplete defecation.” Such a definition allows for diagnosing even mild bowel disfunction. Therefore, additionally, at least one of the following three criteria needs to be met:

1. Ease of defecation assessed as moderate to extreme difficulty (≥2 in (0–4) scale).
2. Last bowel movements present ≥ 2 days before.
3. Frequency of bowel movements reported as ≤ 3 days with defecation per week.

The first criterion is a subjective patient-reported symptom. The rest two symptoms may be reported by a patient and by his/her caregiver, therefore, independent of the patient’s ability to communicate. Moreover, this algorithm allows applying appropriate laxative intervention without unnecessary delay before bowel obstruction develops. We believe the criteria are also easily memorable, which is pivotal for a daily practice (the mnemotechnic acronym ELF-7, which stands for Ease-Last-Difficulty of BM in the past 7 days). As mentioned, this algorithm should be validated in multicenter research in different types of palliative and long-term care settings, in cancer and non-cancer patient populations.

It is noteworthy that results did not differ between subgroups of patients treated with opioid analgesics and those not taking opioids. This reflects the fact that constipation in palliative care patients has multi-factorial etiology, and opioids are only one of a wide variety of causes [1,10,19]. It also suggests that the management of constipation in palliative care should address all concomitant causes. Oral opioids result in a higher frequency of constipation than transdermal forms in numerous studies. However, this has not been confirmed in large retrospective cohort studies in a real clinical setting where various risk factors of constipation are common [20,21]. The correlation of opioid dose and intensity of constipation symptoms was not the goal of this study, and evidence for the impact of opioid dose on constipation frequency is scarce and needs further research.

Our results could apply to other vulnerable populations, such as long-term care patients or nursing home residents, especially those unconscious, although further research is necessary.

The strength of this study is the large sample size and the balanced representation of inpatient, home, and ambulatory palliative care patients, which enabled statistically significant conclusions. Since all questionnaires were filled-in as part of a clinician’s routine work and not as members of a research team, the potential bias seems negligible. The failure in the cross-cultural appraisal of BFI should also be regarded as a limitation of the study, although it revealed the real importance of specific elements. The ECOG scale for assessing general status was used instead of one which may be considered more precise, i.e., Palliative Performance Status [22], or Karnofsky Score. However, neither were validated in the Polish language at that time. A further weakness is a possible underreporting of the use of laxatives and perhaps biased judgment regarding the necessity of their use. Instead of “occasionally” and “often,” a more precise frequency of laxative applications would be more distinctive. The risk is that the all-important subjective patient-reported feeling of the necessity of laxatives would be lost.

## 5. Conclusions

Based on the study results, a definition of constipation in palliative care should consist of both objective and patient-reported measures within a reasonably short period of observation, i.e., seven days. The proposed algorithm for diagnosing constipation includes difficulty in defecation of ≥ 2 on (0–4) scale (moderate to extreme), last BM ≥ 2 days, and frequency of BM ≤ 3 per week. If at least one of these reaches or surpasses the cut-off value, constipation should be diagnosed, and laxative treatment modified. Further research is necessary to assess the feasibility and validity of the algorithm.

## Figures and Tables

**Table 1:** Patient Demographics and Baseline Characteristics.

	Study 1	Study 2	*p*	Total
Number of patients, *n*	248	121		369
Age [years], mean (95% CI)	68.0 (66.5–69.6)	69.1 (67.0–71.3)	0.280	68.4 (67.1–69.6)
Women, *n* (%)	117 (47.2%)	67 (56.8%)	0.086	184 (50.3%)
Palliative care service *n* (%)			**<0.0001**	
Inpatient hospice	128 (51.6%)	28 (23.1%)		156 (42.3%)
Home care	40 (16.1%)	76 (62.8%)		116 (31.4%)
Ambulatory	78 (31.4%)	17 (14.0%)		95 (25.7%)
Unspecified	2 (0.8%)	-		2 (0.5%)
ECOG, mean (95% CI)	2.6 (2.5–2.7)	2.3 (2.0–2.5)	**0.022**	2.5 (2.4–2.6)
The primary site of the tumor, *n* (%)			**0.001**	
Colorectal	10 (4.0%)	14 (11.6%)		24 (6.5%)
Other gastrointestinal	32 (12.9%)	13 (10.7%)		45 (12.2%)
Non-GI cancer	206 (83.0%)	90 (74.4%)		296 (80.2%)
Unspecified	-	4 (3.3%)		4 (1.1%)
Strong opioids, patients *n* (%)	139 (56.0%)	86 (71.1%)	**0.005**	225 (61.0%)
OME [mg/day], mean (95% CI)	86.8 (72.3–101.3)	112.7 (93.6–131.7)	**0.004**	98.9 (87.6–110.3)
Laxative treatment, % (95% CI)				
diet modification	42.3 (36.1–48.5)	44.6 (35.6–53.6)	0.721	43.0 (38.0–48.2)
oral	42.3 (36.1–48.5)	44.6 (35.6–53.6)	0.535	45.3 (40.2–50.4)
suppositories	28.6 (23.0–34.3)	29.8 (21.5–38.0)	0.861	29.0 (24.3–33.6)
enema	11.3 (7.3–15.3)	11.6 (5.8–17.4)	0.966	11.4 (8.1–14.6)
manual evacuation	6.0 (3.0–9.0)	4.1 (0.5–7.7)	0.765	5.4 (3.1–7.7)

ECOG—Eastern Cooperative Oncology Group scale; Non-GI—Non-gastrointestinal; OME—oral morphine equivalent. Bold means significant differences.

**Table 2:** Symptoms of constipation and assessment scales.

Symptom or Assessment Scale	Valid *n*	Mean (95% CI)	Median (Q25–Q75)	Range
Last BM [days]	369	2.4 (2.2–2.7)	2 (1–4)	0–10
Frequency of BM [days/week]	369	2.7 (2.5–2.9)	2 (1–3)	0–7
Difficulty of defecation (0–4)	369	2.2 (2–2.3)	2 (1–3)	0–4
Necessity of laxatives (0–4)	121	1.5 (1.3–1.8)	1 (0–3)	0–4
BFI (0–10)	248	4.5 (4.1–4.9)	4.3 (1.3–7.5)	0–10
Ease of defecation	248	4.8 (4.4–5.2)	5 (2–8)	0–10
Feeling of incomplete bowel evacuation	243	3.8 (3.4–4.2)	3 (1–7)	0–10
Personal judgment of patient regarding constipation	241	4.8 (4.3–5.2)	5 (1–8)	0–10
PAC-SYM (0–4)	211	1.3 (1.2–1.4)	1.3 (0.6–1.8)	0.1–3.8
**Abdominal symptoms**	211	1.3 (1.2–1.4)	1.3 (0.6–1.8)	0.1–3.8
Discomfort	211	1.5 (1.4–1.7)	1 (1–2)	0–4
Pain	210	1.3 (1.1–1.4)	1 (0–2)	0–4
Bloating	211	1.5 (1.4–1.7)	1 (0–3)	0–4
Cramps	210	0.9 (0.8–1.1)	1 (0–2)	0–4
**Stool symptoms**	211	0.7 (0.6–0.9)	0.7 (0–1.3)	0–4
Painful BM	211	1.3 (1.1–1.5)	1 (0–2)	0–4
Rectal burning	210	0.6 (0.5–0.8)	0 (0–1)	0–4
Rectal bleeding	207	0.3 (0.2–0.4)	0 (0–0)	0–4
**Rectal symptoms**	369	1.7 (1.5–1.8)	1.8 (0.6–2.6)	0–4.5
Incomplete	367	1.6 (1.5–1.8)	2 (0–3)	0–4
Too hard	369	1.8 (1.7–2.0)	2 (0–3)	0–4
Too small	368	1.5 (1.4–1.7)	1 (0–3)	0–4
Straining or squeezing	367	2.0 (1.8–2.1)	2 (0–3)	0–4
“False alarm”	236	0.9 (0.8–1.1)	0 (0–2)	0–4

*n*—number; CI—confidence interval; Q25–Q75—quartile 1 to 3 range; BM—bowel movements; BFI—bowel function index; PAC-SYM—Patient Assessment of Constipation Symptoms score.

**Table 3:** Spearman rank-order correlations (bold when *p* < 0.05; bold underlined when *p* < 0.001).

Variable	*n*	Last BM	Frequency of BM	Difficulty of Defecation	Necessity of Laxatives
Age	363	−0.02	0.01	−0.05	0.03
ECOG	355	0.21	−0.15	0.10	0.00
Last BM	369	-	−0.76	0.53	0.14
Frequency of BM	369	−0.76	-	−0.61	−0.20
Difficulty of defecation	369	0.53	−0.61	-	0.37
Necessity of laxatives	121	0.14	−0.20	0.37	-
BFI	248	0.55	−0.67	0.94	n/a
PAC-SYM	211	0.36	−0.51	0.65	n/a
PAC-SYM abdominal	211	0.30	−0.37	0.41	n/a
Discomfort	211	0.31	−0.39	0.47	n/a
Abdominal pain	210	0.22	−0.24	0.20	n/a
Bloating	211	0.23	−0.30	0.45	n/a
Cramps	210	0.20	−0.22	0.17	n/a
PAC-SYM rectal	211	0.23	−0.27	0.33	n/a
Painful bowel movements	211	0.21	−0.28	0.32	n/a
Rectal burning	210	0.28	−0.22	0.18	n/a
Rectal bleeding	207	0.23	−0.09	0.17	n/a
PAC-SYM stool	369	0.49	−0.54	0.75	0.24
Incomplete defecation	367	0.37	−0.41	0.56	0.23
Stools too hard	369	0.46	−0.50	0.64	0.19
Stools too small	368	0.37	−0.42	0.55	0.21
Straining	367	0.44	−0.49	0.66	0.34
“False alarm”	236	0.21	−0.36	0.54	n/a

BM—bowel movements; BFI—bowel function index; ECOG—Eastern Cooperative Oncology Group score.

**Table 4:** The proportion of patients meeting the criteria for constipation.

Criteria	*n*	Frequency (95% CI)
Criterion 1. Ease of defecation ≥ 2 on (0–4) scale (moderate to extreme).	369	65.0% (60.2–69.9%)
Criterion 2. Last BM ≥ 2 days.	369	60.2% (55.1–65.2%)
Criterion 3. Frequency of BM ≤ 3 per week.	369	75.1% (70.6–79.5%)
Criterion 4. Necessity of laxatives (≥1 on (0–4) scale).	121	71.1% (62.9–79.3%)
Criteria 1–4 met		83.5% (79.7–87.3%)
Criteria 1–3 met (without criterion 4)		82.7% (78.8–86.5%)

## Data Availability

The data presented in this study are available on request from the corresponding author for any academic use upon citation of this article. The data are not publicly available due to privacy and permission restricted to publication in the form of an article.
